# Supplementary material for: Imported malaria predominates in near-elimination settings in Southwestern Uganda
Source: PLOS Glob Public Health. 2026 Aug 12;6(8):e0005951. doi: 10.1371/journal.pgph.0005951 (PMC13465794; doi:10.1371/journal.pgph.0005951)
Supplement: Supplementary file 1 [file pgph.0005951.s005.docx]

**Table S1.** **Site-specific prevalence (%) of mixed/mutant genotypes for drug resistance–associated loci in Maziba, Muko, and Kamwezi.**

| **Drug Resistance Mutation** | **Maziba** | **Muko** | **Kamwezi** |
| --- | --- | --- | --- |
| **k13_A578S** | 2.9 | 0.0 | 0.0 |
| **k13_A675V** | 8.7 | 6.8 | 4.0 |
| **k13_R539T** | 0.0 | 1.0 | 0.0 |
| **k13_P441L** | 10.1 | 13.3 | 22.0 |
| **k13_R561H** | 1.4 | 4.8 | 18.0 |
| **k13_C469F** | 4.3 | 1.0 | 7.0 |
| **k13_C469Y** | 2.9 | 1.0 | 0.0 |
| **crt_K76T** | 0.0 | 2.0 | 0.0 |
| **dhfr_C59R** | 98.6 | 100.0 | 100.0 |
| **dhfr_I164L** | 55.1 | 61.9 | 54.0 |
| **dhfr_N51I** | 100.0 | 100.0 | 100.0 |
| **dhfr_S108N** | 100.0 | 100.0 | 100.0 |
| **dhps_A581G** | 47.8 | 42.9 | 42.0 |
| **dhps_G437A** | 0.0 | 1.9 | 2.0 |
| **dhps_K540E** | 100.0 | 100.0 | 99.0 |
| **mdr1_D1246Y** | 2.8 | 9.6 | 4.0 |
| **mdr1_Y184F** | 69.5 | 61.9 | 40.0 |
